# Short Stem vs. Standard Stem in Primary Total Hip Replacement: A Perioperative Prospective Invasiveness Study with Serum Markers

**DOI:** 10.3390/diseases13080233

**Published:** 2025-07-23

**Authors:** Marco Senarighi, Carlo Ciccullo, Luca de Berardinis, Leonard Meco, Nicola Giampaolini, Simone Domenico Aspriello, Luca Farinelli, Antonio Pompilio Gigante

**Affiliations:** 1 Maria Cecilia Hospital—GVM Care & Research, 48033 Cotignola, Italy; senarighi.m@gmail.com; 2 Clinical Ortopaedics, Department of Clinical and Molecular Sciences, Politecnica University of Marche, 60126 Ancona, Italy; leonard.meco@ospedaliriuniti.marche.it (L.M.); nicola.giampaolini@ospedaliriuniti.marche.it (N.G.); farinelli.luca92@gmail.com (L.F.); a.p.gigante@staff.univpm.it (A.P.G.); 3 IRCCS Ospedale San Raffaele, Unità di Ortopedia e Traumatologia, Via Olgettina 60, 20132 Milan, Italy; luca8191deberardinis@gmail.com; 4 ASD Dental Clinic, 61121 Pesaro, Italy; simonedomenico@yahoo.it; 5 IRCCS Istituto Nazionale Ricovero e Cura per Anziani (INRCA), 60126 Ancona, Italy

**Keywords:** total hip arthroplasty, serum markers, posterolateral approach, short stem

## Abstract

Background: Total hip arthroplasty (THA) is a well-established surgical procedure for end-stage hip arthrosis. Innovations such as minimally invasive approaches and new technologies have improved outcomes and reduced invasiveness. The introduction of short-stem prostheses, which offer potential benefits in bone preservation, has been a significant development in recent years. This prospective case series study aims to compare invasiveness of the short-stem (SS) and conventional-stem (CS) prostheses in THA with a posterolateral approach (PLA) by assessing perioperative serum markers. Methods: A prospective case series was conducted involving consecutive patients who underwent primary THA from January 2022 to December 2023. Demographics and preoperative, postoperative day 1 (POD1), and postoperative day 2 (POD2) serum levels of C-reactive protein (CRP), erythrocyte sedimentation rate (ESR), procalcitonin (PCT), and white blood cells (WBCs) were measured. Results: The study included 21 patients with CS and 19 with SS, with no significant differences between groups in demographic. No statistically significant differences were found in serum markers between SS and CS groups at any time point. Both groups showed significant increases in ESR, CRP, and PCT from preoperative levels to POD2 (*p* < 0.001), while WBC values increased from preoperative to POD1 but decreased between POD1 and POD2. Conclusion: The short-stem prosthesis does not exhibit significantly different perioperative serum marker profiles compared to the conventional stem, suggesting similar levels of surgical invasiveness between the two implants. Further studies with larger sample sizes are needed to validate these findings and explore other aspects of short-stem THA.

## 1. Introduction

Total hip arthroplasty (THA) has become a standard surgical intervention for patients with hip joint disorders [1]. The incidence of THA has increased rapidly over the last decade due to both the ageing population in Western societies and increasingly sedentary lifestyles. Hip arthroplasty is no longer reserved only for the elderly; advancements in biomedicine and surgical techniques have made it a viable solution for younger patients who are part of the working population [2,3,4].

Numerous studies have demonstrated the efficacy and safety of THA in relieving pain, improving function, and enhancing quality of life for patients with hip joint disorders [5,6]. Recent years have seen significant advances in surgical techniques for hip arthroplasty, including minimally invasive approaches such as the direct anterior approach, which is associated with reduced blood loss and shorter hospital stays [7,8]. Additionally, the advent of new technologies such as computer-assisted and robot-assisted surgery has shown promising results in improving the accuracy and precision of implant placement [9,10]. The design of implants has also evolved, aiming to improve the survival and performance of THA while minimizing invasiveness.

Since Dr. Charnley’s introduction of THA in 1962, the general concepts have remained largely unchanged. Charnley’s arthroplasty was characterized by a one-piece stainless steel femoral stem, high-density polyethylene, self-curing polymethyl methacrylate, and a stainless steel framework [11]. Although the basic principles of arthroplasty have remained remarkably similar, the design of the implants has evolved over the past 50 years.

With the changing patient demographics, bone preservation has become essential and proximal fixation with less subsequent stress shielding has become a focal point. Short-stem THA has become increasingly popular in recent years, including short metaphyseal stems without distal extension, addressing issues of diaphyseal diameter misalignment seen with double-taper stems [12]. Some controversy remains over its use in high-risk categories, such as osteoporotic elderly women [13,14,15]. In fact, short stems have been accepted for use in younger patients [16]. This is because young patients may benefit from increased bone preservation in case of possible future revisions [15,17,18].

One of the main factors complicating the evaluation of the literature on short stems is the changing definition of what constitutes a short stem and which implants meet this definition. Feyen and Shimmin’s classification focused on stem length and fixation in bone [19]. Falez et al.’s classification is based on four categories of prostheses according to the cut of the neck [20].

Recent research suggests that serum enzyme levels could serve as objective biomarkers for assessing muscle damage, inflammation, and perioperative stress resulting from surgical interventions [21,22].

This study aims to evaluate whether there is a difference in surgical invasiveness between short stems (SSs) and standard (or conventional, CSs) stems, as measured by serum markers (CRP, ESR, PCT, and WBCs) in the perioperative time, in patients undergoing primary hip arthroplasty with a posterolateral approach (PLA).

## 2. Materials and Methods

A prospective case series was designed, recruiting consecutive patients undergoing total hip arthroplasty for primary hip osteoarthritis, treated by the senior authors (A.G. and N.G.) at “AOU delle Marche-Ancona” between January 2022 and December 2023. All patients signed informed consent to participate in the study and to allow the use of clinical data for research purposes. The study was approved by the Internal Review Board of the Polytechnic University of Marche, (ID 1825 n.90/2021—approved on 5 March 2021.), Ancona, Italy. The study was conducted in accordance with the ethical principles outlined in the Declaration of Helsinki.

Prior to surgery, patients underwent a dental examination.

Data collected included demographics, body mass index (BMI), medical history, current medications, preoperative diagnosis, inpatient history, American Society of Anesthesiologists (ASA) classification, type of anesthesia, pre- and postoperative serum values of CRP, ESR, PCT, and WBCs, any intraoperative complications, and any infections occurring within 3 months after surgery.

### 2.1. Inclusion and Exclusion Criteria

All patients were diagnosed for primary hip osteoarthritis and consecutively underwent the THA procedure using a PLA with implantation of a dual-mobility Sunfit TH cup (Serf, France) and Hype or Hype Mini femoral stems (Serf, France). They are made of titanium alloy (TA6V) and surface-treated with titanium spray and hydroxyapatite (HAP). The Hype Mini stems have a 20% shorter intramedullary component. All stems in the Hype range require femoral neck resection. Patients enrolled were aged 18–70 years. Exclusion criteria were unwillingness to participate; a BMI ≥ 35; inflammatory arthropathy and autoimmune disease; previous hip and/or knee surgery; bilateral THA; congenital or acquired muscle disease; ischemic heart disease; end-stage renal failure; a history of hepatitis, liver disease, or malignant liver tumor; peripheral neuropathy; treatments with immunosuppressive or myotoxic drugs; infections in the perioperative period (respiratory/urinary); presence of periodontitis [23]; intraoperative surgical/clinical complications; COVID-19 infection or contact with infected persons during hospitalization; early postoperative infections (first 3 months); postoperative complications; fever during hospitalization; and perioperative transfusions.

### 2.2. Surgical Approach

All procedures were performed using a standard posterolateral approach, with the patient in the lateral decubitus position. After standard skin preparation and draping, a curved skin incision was made, centered over the posterior aspect of the greater trochanter, extending proximally and distally along the femoral shaft. The fascia lata and gluteal fascia were incised in line with the skin incision. The fibers of the gluteus maximus muscle were bluntly split to expose the underlying short external rotators.

The piriformis tendon and conjoined tendons (obturator internus and gemelli) were identified, tagged with non-absorbable sutures, and detached near their insertion to allow for posterior capsulotomy. The posterior capsule was incised and not repaired. After posterior dislocation of the femoral head, acetabular and femoral preparation was performed following standard protocols for total hip arthroplasty, depending on the underlying pathology (displaced femoral neck fracture or advanced coxarthrosis). At the end of the procedure, the short external rotators were reattached to the femur using the preplaced sutures to enhance soft tissue stability and reduce the risk of dislocation.

### 2.3. Perioperative Procedures

All patients received perioperative antibiotic prophylaxis with 2 g of cefazolin. A 16-inch subfascial suction drain was routinely placed, and it was removed 24 h after surgery. Before removal, a meticulous asepsis of the skin was performed with a 10% aqueous povidone–iodine solution. Postoperative pain control included 20 mg of intravenous tramadol and 10 mg of metoclopramide for 24 h. Over the next few days, oral paracetamol or oral tramadol were administered as needed. Postoperative thrombo-embolic prophylaxis was performed with a low-molecular-weight heparin and the use of elastic stockings (both limbs). Physiotherapy, weightbearing, and walking with aids were allowed on POD1.

### 2.4. Serum Markers

As the aim of this present study was to compare serum markers to evaluate differences in femoral stem implantation invasiveness, white blood cells (WBCs), C-reactive protein (CRP), erythrocyte sedimentation rate (ESR), and procalcitonin (PCT) were mined from routinary blood samples performed preoperatively, on the first postoperative day (POD1) and on the second postoperative day (POD2). For each serological marker, mean and standard deviation values were calculated on preoperative day, POD1, and POD2 for both groups (CS and SS).

### 2.5. Statistical Analysis

All analyses were conducted using Microsoft Excel (Microsoft) with the XLSTAT resource pack (XLSTAT-Premium, Addinsoft, New York, NY, USA). The Shapiro–Wilk test was performed to assess whether the data showed a normal distribution. A non-parametric test (Mann–Whitney for unpaired data) was applied to assess continuous variables for significant differences between the groups. The categorical data were subjected to Fischer’s exact test or a chi-square test. The non-parametric Friedman test for repeated measures was carried out to assess variation in serum markers over time, using Bonferroni correction for repeated measures. A *p* value < 0.05 was considered significant.

## 3. Results

The resulting sample consisted of 40 patients (M:F = 1:1, with no statistical differences between CS and SS groups, *p* = 0.20), of whom 21 were operated on with conventional stems (CSs) and 19 with short stems (SSs). Moreover, statistical analysis revealed no statistically significant differences between the two groups for age (68.60 ± 17.85 and 72.00 ± 12.19), operated side, BMI, and ASA score. All 40 patients were operated on under peripheral anesthesia, and none were cemented (Table 1).

### Serum Markers

For each of the considered markers (CRP, ESR, PCT, and WBCs), there were no statistically significant differences in the preoperative period between the two groups.

Moreover, no statistically significant differences were found between the two groups at POD1 and POD2 (Table 2).

The statistical analysis for repeated measures reported a significant increase in ESR, CRP, and PCT values for both groups between the preoperative and POD2 (*p* < 0.001).

On the other hand, WBC values were increasing from preoperative to POD1 but tended to decrease between POD1 and POD2 (Table 3). No statistically significant differences were found in short-stem group between POD1 and POD2 measurement (*p* > 0.05).

## 4. Discussion

The most important finding in this study was that there were no statistically significant differences between the two groups on POD1 and POD2 for any of the considered markers. In fact, in the analyzed data, there do not appear to be differences in surgical invasiveness between the two stems.

This result is credible, as the short stem’s smaller dimensions and lighter weight may imply decreased stress on the body during implantation. However, the variations in surgical technique and implantation approach between the two stems are minimal, especially in relation to the femoral neck resection procedure that is standard for both implants.

C-reactive protein (CRP) and erythrocyte sedimentation rate (ESR) are inexpensive and non-invasive tests that are often obtained in subjects with orthopedic implants to assess the presence of implant-associated infections. CRP and, to a lesser extent, ESR have proven useful in the diagnosis of hip and knee prosthetic infections [24,25,26,27,28,29,30,31,32]. ESR was determined using the Westergren method. The mean ESR increase was nearly negligible between the preoperative and the first postoperative day and modest between the first and second days: the mean value remained moderate for both groups even on POD2 (≤25 mm/h). This result can be explained by the fact that ESR is a relatively slow-rising inflammatory marker, taking several days to reach high values. CRP has been proposed as a measure of the overall invasiveness of surgical procedures, particularly of tissue damage and perioperative stress [33,34,35,36]. The mean CRP increase was significantly more pronounced for both groups, especially between POD1 and POD2. The higher sensitivity of CRP to inflammatory insults compared to ESR allowed a noticeable variation within approximately 48 h post surgery.

A postoperative elevation in proinflammatory cytokines due to surgical trauma and the healing process is part of the natural course after surgical interventions [37]. Procalcitonin (PCT) is a useful surrogate marker for the differentiation of postoperative infection and unspecific inflammatory reactions after surgery. Procalcitonin (PCT) is widely used as a diagnostic marker for sepsis and systemic inflammatory response syndrome (SIRS) and has proven to be a more accurate marker in the detection of early postoperative infection [37,38,39].

The mean PCT evaluation showed modest increases in both groups. The slight increase observed is likely due to surgical stress. The mean WBC value peaked on POD1 and then tended to normalize on POD2: surgical stress likely led to the release of cytokines and inflammation mediators, affecting bone marrow and increasing circulating white blood cells. By the second day, this acute response was already resolving.

Bouaicha et al. showed in their recent study that, in the case of THR, PCT levels showed a uniform low-level trend with a peak on the second postoperative day. On day 5, values returned almost to preoperative levels. In contrast, CRP levels remained elevated throughout the observation period. Only IL-6 levels showed a peak on the first postoperative day, with a rapid and uniform return to preoperative levels [40].

It is important to note that the term “minimally invasive” does not refer solely to the surgical approach or the size of the implanted components. Rather, it also encompasses factors such as bone stock preservation, revision potential, and the rate of mechanical complications. Although our study focused exclusively on biological aspects, these dimensions warrant further investigation in future longitudinal studies.

This study is, to our knowledge, the first to analyze serum biomarkers in relation to two stem types and their associated surgical invasiveness. A key strength of our research is that all analyses were conducted by the same team at a single institution, utilizing a consistent surgical technique. Other significant advantages are the application of exacting inclusion and exclusion criteria, which ensured a homogeneous patient sample.

However, the present study has several limitations that must be acknowledged. Primarily, the limited sample size and follow-up may have underpowered the study. Secondly, the choice to examine only ESR, CRP, PCT, and WBCs, while omitting other serum markers like interleukins, myoglobin, and tumor necrosis factor alpha, could have contributed to the inability to detect a difference between the two stems.

## 5. Conclusions

The short-stem prosthesis presents a viable option for patients, particularly younger individuals with high functional demands, undergoing total hip arthroplasty due to its advantages in terms of bone preservation and reduced bone loss. However, based on the available data, it cannot yet be classified as a minimally invasive alternative to conventional-stem prostheses. Further research is needed, including studies with larger sample sizes and evaluations of various short-stem designs, to elucidate any differences in surgical invasiveness between short and conventional stems.

## Figures and Tables

**Table 1:** Preoperative and perioperative data of the THA patients.

Variable	Conventional Stem (n = 21)	Short Stem (n = 19)	*p-Value*
Age, mean (SD)	68.60 (17.85)	72.00 (12.19)	0.20
Gender			
Male (%)	8 (38)	12 (63)	0.20
Female (%)	13 (62)	7 (37)	
Side:			
Right (%)	7 (33)	4 (21)	0.49
Left (%)	14 (67)	15 (79)	
BMI (kg/m^2^), mean (SD)	23.03 (1.66)	22.68 (1.89)	0.52
ASA class (%)			
ASA 1	4 (19)	4 (21)	0.99 ^§^
ASA 2	9 (43)	8 (42)	
ASA 3	8 (38)	7 (37)	
Surgical time (min), mean (SD)	56.5 (29.52)	53.5 (10.96)	0.84
Anesthesia			
General (%)	0	0	1.00
Spinal (%)	21 (100)	19 (100)	
Cementation			
Yes (%)	0	0	1.00
No (%)	21 (100)	19 (100)	

ASA: American Society of Anesthesiology; SD: standard deviation. ^§^ chi-square test.

**Table 2:** Comparison between CS and SS group.

Variable	Conventional Stem (*n = 21*)	Short Stem (*n = 19*)	*p-Value*
*ESR (mm/h)*			
Preoperative, mean (SD) (range)	11.80 (5.91) (4.00–19.00)	8.68 (4.80) (5.00–19.00)	0.14
POD1, mean (SD) (range)	12.80 (8.06) (4.00–22.00)	11.37 (6.73) (4.00–21.00)	0.59
POD2, mean (SD) (range)	25.20 (17.37) (5.00–51.00)	22.68 (11.45) [6.00–35.00)	0.97
*CRP (mg/dL)*			
Preoperative, mean (SD) (range)	1.10 (1.40) (0.10–3.73)	0.79 (0.56) (0.15–1.37)	0.91
POD1, mean (SD) (range)	8.65 (3.18) (3.45–11.7)	8.97 (4.89) (2.46–14.94)	0.38
POD2, mean (SD) (range)	17.07 (3.91) (12.16–28.00)	17.76 (5.52) (7.2–21.76)	0.32
*PCT (ng/mL)*			
Preoperative, mean (SD) (range)	0.03 (0.01) (0.01–0.05)	0.04 (0.03) (0.01–0.10)	0.20
POD1, mean (SD) (range)	0.36 (0.17) (0.10–0.56)	0.27 (0.17) (0.07–0.53)	0.09
POD2, mean (SD) (range)	0.45 (0.30) (0.19–1.04)	0.35 (0.21) (0.07–0.69)	0.47
*WBC* (×*10^9^ L*)			
Preoperative, mean (SD) (range)	7.33 (1.45) (5.24–9.62)	7.87 (1.09) (5.59–10.24)	0.09
POD1, mean (SD) (range)	11.19 (2.33) (8.84–15.89)	11.83 (3.75) (7.12–16.38)	0.67
POD2, mean (SD) (range)	10.31 (2.00) (5.87–13.87)	11.18 (3.40) (7.04–16.15)	0.16

ESR: erythrocyte sedimentation rate; CRP: C-reactive protein; PCT: procalcitonin; WBC: white blood cell; POD: postoperative day; SD: standard deviation.

**Table 3:** Variation of serum markers over time in CS and SS group.

Conventional Stem (n = 21)				
Variable	Preoperative, Mean (SD)	POD1, Mean (SD)	POD2, Mean (SD)	*p-Value*
*ESR (mm/h)*	11.80 (5.91)	12.80 (8.06)	25.20 (17.37)	<0.001
*CRP (mg/dL)*	1.10 (1.40)	8.65 (3.18)	17.07 (3.91)	<0.001
*PCT (ng/mL)*	0.03 (0.01)	0.36 (0.17)	0.45 (0.30)	<0.001
*WBC* (×*10^9^/L*)	7.33 (1.45)	11.19 (2.33)	10.31 (2.00)	<0.001
**Short Stem (n = 19)**				
**Variable**	**Preoperative, Mean (SD)**	**POD1, Mean (SD)**	**POD2, Mean (SD)**	***p-Value***
*ESR (mm/h)*	8.68 (4.80)	11.37 (6.73)	22.68 (11.45)	<0.001
*CRP (mg/dL)*	0.79 (0.56)	8.97 (4.89)	17.76 (5.52)	<0.001
*PCT (ng/mL)*	0.04 (0.03)	0.27 (0.17)	0.35 (0.21)	<0.001
*WBC* (×*10^9^/L*)	7.87 (1.09)	11.83 (3.75)	11.18 (3.40)	0.029 n.s.

ESR: erythrocyte sedimentation rate; CRP: C-reactive protein; PCT: procalcitonin; WBC: white blood cell; POD: postoperative day; SD: standard deviation.

## Data Availability

The data presented in this study are available on request from the corresponding author. The data are not publicly available due to privacy.
